# Plasticity of myeloid-derived suppressor cells in cancer and cancer therapy

**DOI:** 10.32604/or.2025.060063

**Published:** 2025-06-26

**Authors:** JIAJIA LV, XIAOYOU ZHONG, LIN WANG, WEIFEI FAN

**Affiliations:** Department of Hematology and Oncology, Geriatric Hospital of Nanjing Medical University, Jiangsu Province Geriatric Hospital, Nanjing, 210000, China

**Keywords:** Tumor microenvironment (TME), Differentiation, Dendritic cells, Macrophages

## Abstract

The tumor microenvironment (TME) is a complex and dynamic network comprised of tumor cells, surrounding cellular components, various signaling molecules, and the stroma. Myeloid-derived suppressor cells (MDSCs) are pivotal players in the immunosuppressive landscape of the TME, effectively hindering antitumor immune responses and facilitating tumor progression. Originating from pathologically activated myeloid precursors and relatively immature myeloid cells, MDSCs retain plasticity to further differentiate into other myeloid cells, such as macrophages or dendritic cells, which underpins their heterogeneity and adaptability in response to the TME. In this review, we delve into the plasticity of MDSCs in the tumor microenvironment and illuminate the underlying mechanisms that enable them to modulate immune responses. Furthermore, we explore the implications of MDSCs plasticity for cancer therapy, particularly its role in enhancing the efficiency of combination treatments.

## Introduction

Hematopoietic stem cells typically undergo a series of processes to differentiate into mature cells, including macrophages, dendritic cells, and granulocytes [1]. Myeloid differentiation constitutes the primary mechanism of host protection. The classical activation of mature myeloid cells is driven by danger-associated molecular patterns (DAMPs), pathogen-associated molecular patterns (PAMPs), and Toll-like receptors (TLRs), resulting in a robust and temporary response. In the hypoxic, acidic, and high reactive oxygen species (ROS) tumor microenvironment (TME), tumor-derived factors provide a continuous stimulus that alters myeloid differentiation, leading to the generation of immunosuppressive cell subsets [2]. The accumulation of unfolded proteins in the endoplasmic reticulum (ER) triggers ER stress, which drives the pathological activation of the immunosuppressive phenotype in myeloid-derived suppressor cells (MDSCs) [3,4]. MDSCs can be classified into two subtypes: monocytic MDSCs (M-MDSCs) and polymorphonuclear MDSCs (PMN-MDSCs).

The definition of MDSCs remains controversial. MDSCs are not a single actual myeloid subset but rather a heterogeneous population of myeloid cells, consisting of pathologically activated myeloid precursors and relatively immature myeloid cells with immunosuppressive properties [4,5]. Recent studies suggest that not all MDSCs are undifferentiated cells. For example, Francesca Pettinella et al. identified mature PMN-MDSCs that retain immunosuppressive functions in the TME, regulating T and Natural Killer cell (NK cell) activity to promote tumor immune evasion [6]. This underscores the diverse roles of MDSCs in tumor immunity, which are influenced by their differentiation status and the surrounding settings. Mouse CD11b^+^Gr-1^+^ myeloid cells were first found immunosuppressive and defined according to cell-surface markers [7]. Based on the expression of Ly6C and Ly6G, M-MDSCs were defined as CD11b^+^Ly6G^lo^Ly6C^hi^, and PMN-MDSCs were defined as CD11b^+^Ly6G^hi^Ly6C^lo^. Moreover, Ly6C^hi^ monocytes are often regarded as pro-inflammatory monocytes and share a common phenotype with MDSCs. Many studies have shown that Ly6C^hi^ monocytes have immunosuppressive activity and function as a subset of MDSCs within the TME [2].

MDSCs exhibit significant plasticity, allowing them to adapt their phenotype and function, and differentiate into various immune cell subsets, depending on environmental cues such as hypoxia, cytokines, and inflammation within the tumor microenvironment [8]. This inherent flexibility allows MDSCs to respond to changes within the TME and participate in a variety of immune suppression processes. MDSCs can differentiate into mature myeloid cells, such as tumor-associated macrophages (TAMs) and dendritic cells (DCs), as well as osteoclasts [9], fibroblasts [10], or phenotypes that activate CD8^+^ T cells through stimulator of interferon genes (STING)-dependent induction of type I interferons (IFNs) [11]. Recent studies have highlighted the differentiation of MDSCs into TAMs and DCs. This review discusses the plasticity of MDSCs in cancers and their role in cancer therapy, particularly in the context of targeting MDSCs and combination treatments.

## Characteristics of MDSCs

### Differentiation, recruitment, and activation of MDSCs

The generation of MDSCs occurs in two main stages: expansion and activation. Typically, MDSCs expand in the bone marrow or spleen and are activated within the TME, where they exert immunosuppressive effects. These two stages may partially overlap [2]. In both tumor-bearing mice and cancer patients, MDSCs exhibit a functional gradient, with less suppression in the bone marrow and more potent ones in the spleen, blood, and tumor. This progression highlights the critical role of the TME in activating and differentiating MDSCs, enhancing their immunosuppressive activity [12].

The differentiation of MDSCs is mainly regulated by signal transducer and activator of transcription3 (STAT3), CCAAT/enhancer-binding protein β (C/EBPβ), and interferon regulatory factor 8 (IRF8). STAT3 is a major regulator of MDSCs differentiation via the CD39/CD73-adenosine pathway [13]. Conversely, STAT3 inhibition can induce MDSCs apoptosis. V-domain suppressor of T cell activation (VISTA), a major factor in regulating myeloid differentiation, promotes MDSCs differentiation by maintaining STAT3 activation and polyamine synthesis which activates the JAK/STAT3 pathway by enhancing the activity of casein kinase 2 [14]. Crucial in myeloid differentiation, IFR8 mainly promotes monocyte-DCs differentiation and impresses immature myeloid cell differentiation to PMN-MDSCs [15]. Highly expressed C/EBPβ encourages the expansion of MDSCs by activating factors such as interleukin-6 (IL-6), colony stimulating factors (CSFs), CSFRs, and Matrix Metalloproteinases (MMPs) [16]. In addition, cytokines such as granulocyte-macrophage colony-stimulating factor (GM-CSF), granulocyte colony-stimulating factor (G-CSF), IL-1β, IL-4, IL-6, IL-13, tumor necrosis factor (TNF), IFN-γ, and vascular endothelial growth factor (VEGF), also promote the accumulation of MDSCs. These cytokines act as inducers of the above signaling pathways [2,12].

Bone marrow MDSCs are mainly recruited into the TME via the CCL2/CCL12-CCR2, CCL3/4/5-CCR5, CCL15-CCR1, and so on pathways [17,18]. The TME is hypoxia, adenosine accumulation, low pH, and low tryptophan. Hypoxia recruits MDSCs and inhibits T cells and NK cells in the TME. The acid environment caused by hypoxia not only favors tumor cell survival and metastasis but also affects the activity and function of other immune cells [19,20]. Multiple factors contribute to MDSCs activation, including IFN-γ, IL-1β, IL-4, IL-13, GM-CSF, prostaglandin E_2_ (PGE_2_), cyclooxygenase2 (COX2), STAT1, STAT6, and nuclear factor κB (NF-κB). Activated MDSCs promote tumor angiogenesis and metastasis, and cooperate with or transform into other immune cells [21].

The differentiation, recruitment, and activation of MDSCs form the basis for their immunosuppressive functions. MDSCs can further differentiate into mature myeloid cells, such as TAMs and DCs. While these immune cells are essential components of the cellular immune response, their functions tend to lean to immunosuppression in this environment [22].

### Function of MDSCs in cancer immunosuppression

**Figure 1:**
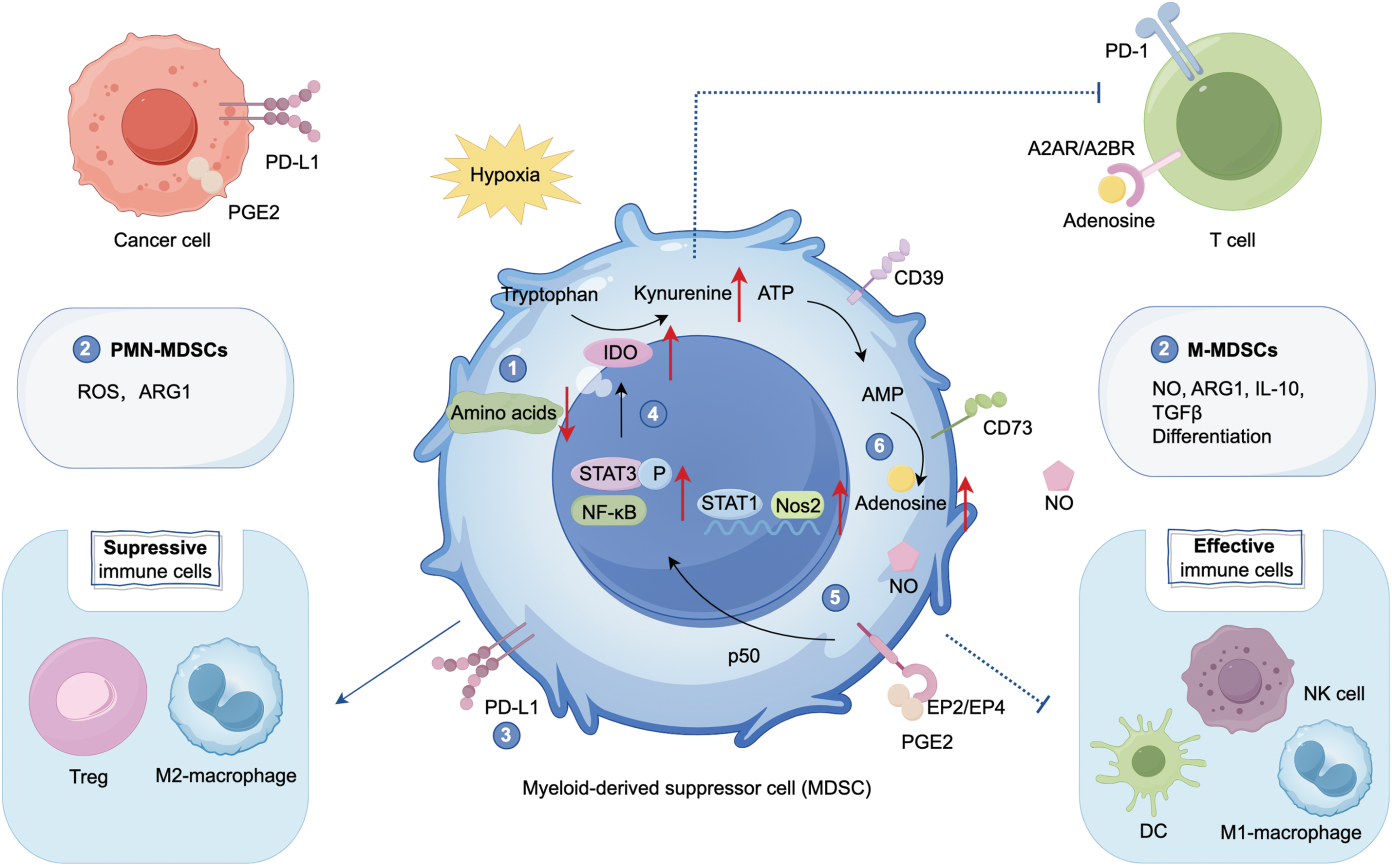
Function of myeloid-derived suppressor cells (MDSCs) in cancer immunosuppression. MDSCs play a pivotal role in the tumor microenvironment (TME) by suppressing effective immune cells such as Natural Killer cells (NK cells), M1 macrophages, and dendritic cells (DCs) and cooperating with other immunosuppressive cells, including Regulatory T cells (Tregs) and M2 macrophages, with T cells serving as the primary target. MDSCs suppress T cell activity through the following mechanisms: (1) depletion of amino acids, (2) secretion of cytokines that inhibit T cell proliferation and function: Polymorphonuclear MDSCs (PMN-MDSCs) primarily secrete reactive oxygen species (ROS) and arginase1 (ARG1), while monocytic MDSCs (M-MDSCs) secrete NO, ARG1, IL-10, and TGFβ, (3) expression of PD-L1, (4) activation of STAT3 and NF-κB signaling pathways, leading to the release of indoleamine 2,3-dioxygenase (IDO), which promotes the conversion of tryptophan to kynurenine, (5) promotion of nitric oxide (NO): Tumor-derived prostaglandin E2 (PGE2)-mediated induction of nuclear p50 nuclear factor κB (NF-κB) facilitates signal transducer and activator of transcription 1 (STAT1) binding to the regulatory regions of IFNγ-dependent genes, such as inducible nitric oxide synthase (Nos2), (6) upregulation of CD39/CD73, facilitating ATP dephosphorylation to adenosine. Adenosine can bind to A2A receptor (A2AR) and A2BR receptors on T cells, suppressing their activity and activating Tregs. Created with FigDraw.

In the TME, MDSCs secrete soluble factors such as arginase1 (ARG1), inducible NO synthase (iNOS), nitric oxide (NO), ROS, transforming growth factorβ (TGFβ), adenosine, IL-10, and PGE_2_ to inhibit T cells and NK cells activity, and suppress antitumor immunity, leading to tumor progression [2,23]. COX2 catalyzes the conversion of arachidonic acid (AA) to PGE_2_. In myeloid cells, PGE_2_ enhances the immunosuppressive functions of MDSCs by activating E-prostanoid receptors EP2 and EP4 [24]. Through the p50 NF-κB signaling pathway, tumor-derived PGE_2_ facilitates the binding of STAT1 to the regulatory regions of IFNγ-dependent genes, such as inducible nitric oxide synthase (Nos2), thereby promoting NO production and contributing to immune suppression [25]. MDSCs facilitate the transformation of ATP to adenosine by expressing ectonucleotidases (CD39 and CD73). Adenosine induces the differentiation of MDSCs, indirectly destroys the cytotoxicity of T cells and NK cells, and activates Regulatory T cells (Tregs) through binding to A2A receptor (A2AR) and A2BR [13]. MDSCs can deplete nutrients like cysteine, L-arginine, and tryptophan, thereby blocking T cell activation and function [20]. MDSCs promote the release of indoleamine 2,3-dioxygenase (IDO) by activating the STAT3 and NF-κB pathways [26,27]. IDO can produce immunosuppressive metabolite kynurenine, thereby inhibiting T cells, NK cells, and DCs, and promoting M2 polarization (Fig. 1).

MDSCs exhibit significant plasticity throughout tumor progression and in response to therapeutic stress, adapting their phenotype and function to the TME. PMN-MDSCs are key mediators of immune suppression, promoting T-cell tolerance via ROS and ARG1. In contrast, M-MDSCs secrete immunosuppressive factors like NO, ARG1, IL-10, and TGFβ, inhibiting both antigen-specific and non-specific T-cell responses. Moreover, M-MDSCs, similar to monocytes, can differentiate into other immune cells, enhancing their immune modulatory functions [28].

## Plasticity of MDSCs in Cancer

### Differentiation of MDSCs into TAMs

Macrophages are vital to both innate immunity and adaptive immunity. They are highly plastic and adaptable in their functions. The phenotypes of macrophages remain challenging to distinguish. Therefore, most studies use the concept of polarization to divide them into two extremes: M1 macrophages and M2 macrophages [29]. However, it is still insufficient to differentiate macrophages only by polarization. Like a color palette, different combinations of spectral patterns give rise to various macrophage subtypes. M-MDSCs can be distinguished from TAMs by higher F4/80 and CSF receptor CD115 expression, lower to moderate Ly6C, and reduced or undetectable S100A9 and IRF8 levels [7]. Single-cell RNA sequencing (scRNA-seq) analysis revealed the presence of cells expressing markers for M-MDSCs, such as IL10, CD14, and VEGFA, as well as markers for PMN-MDSCs, including IL6, oxidized low-density lipoprotein receptor 1 (OLR1), and TGFB1, during the transition from monocytes to M2-like cells. These findings highlight that MDSCs are molecularly distinct from M1/M2 macrophages [30]. Specially, MDSC-derived macrophages have stronger immunosuppressive functions, whereas monocyte-derived macrophages (mo-Macs) tend to exhibit limited immunosuppressive activity, possibly related to S100A8/A9 [22,31].

**Figure 2:**
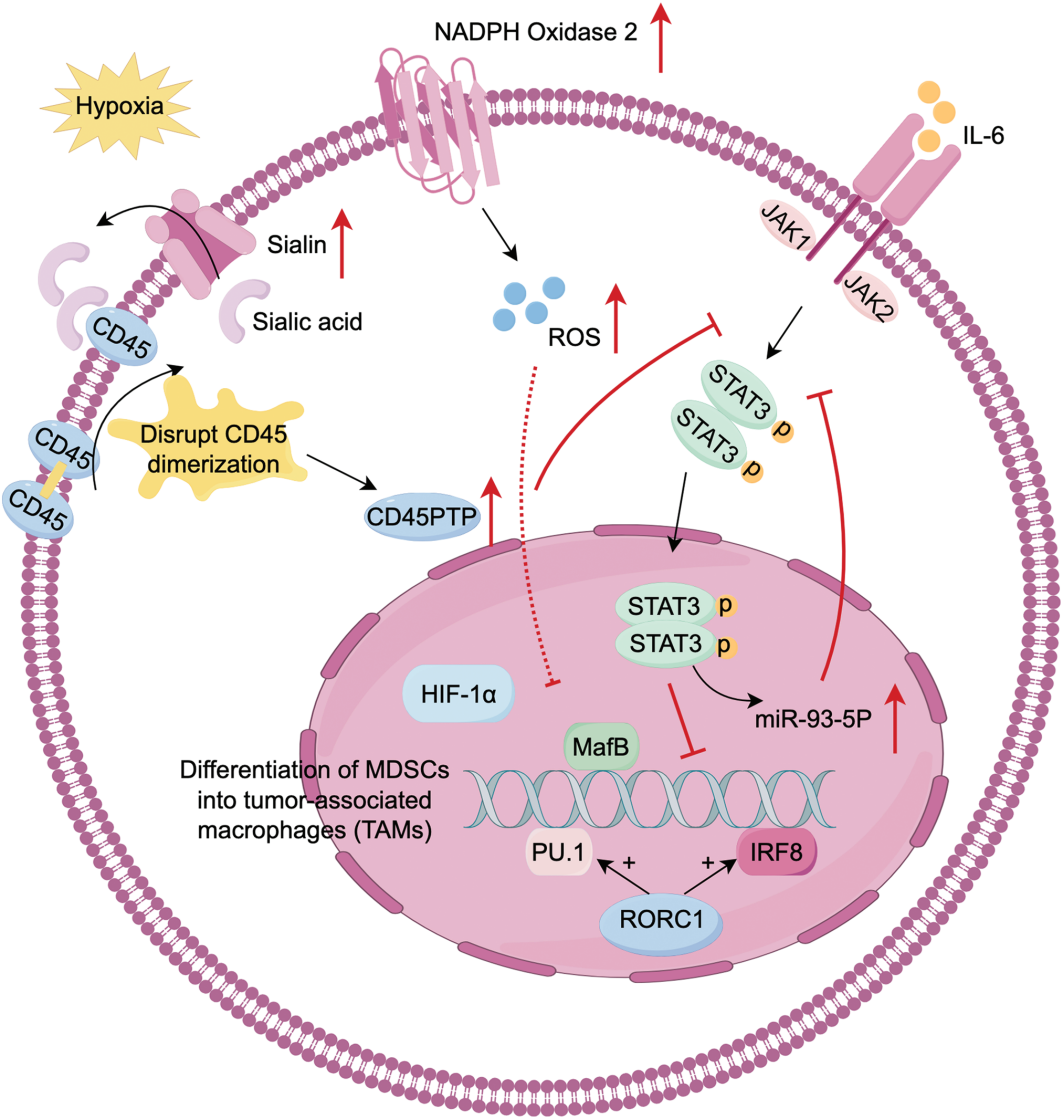
Differentiation of MDSCs into tumor-associated macrophages (TAMs). MDSCs plasticity is strongly influenced by the hypoxic TME, with STAT3 downregulation serving as a prerequisite for differentiation. (1) In hypoxia conditions, MDSCs upregulate sialin, facilitating the transport of sialic acid to the cell surface. Sialic acid binds with CD45 and disrupt CD45 dimerization. These activates CD45 protein tyrosine phosphatase (CD45PTP), leading to STAT3 dephosphorylation. (2) IL-6/JAK/STAT3 produce miR-93-5p, which inhibits STAT3 phosphorylation. (3) IRF8, PU.1, and musculoaponeurotic fibrosarcoma oncogene homolog B (MafB) are key transcription factors driving myeloid progenitor differentiation to the monocytic/macrophage lineage. Retinoic-acid-related orphan receptor (RORC1), a driver of emergency hematopoiesis in cancer, enhances IRF8 and PU.1 expression. (4) Extracellular reactive oxygen species (ROS) promotes monocyte differentiation and M2 polarization, but high ROS levels from NADPH oxidase 2 (NOX2) in MDSCs inhibit their differentiation into TAMs. Created with FigDraw.

The differentiation of MDSCs into macrophages is tissue-dependent. MDSCs differentiate into macrophages in mouse spleen, whereas they rapidly differentiate into TAMs in tumor tissue. This process is regulated by hypoxia which functions in the development of MDSCs and TAMs. In the TME, hypoxia activates CD45 protein tyrosine phosphatases (CD45PTP), which inhibit STAT3 and promote the differentiation of MDSCs to TAMs [32]. STAT3 promotes the amplification of MDSCs. IL-6 activates the JAK/STAT3 pathway, leading to the induction of PMN-MDSCs to produce and secrete miR-93-5p. This miRNA subsequently inhibits STAT3, drives the differentiation of M-MDSCs into M2 macrophages, and accelerates the transition from colitis to cancer [33]. This indicates that STAT3 dephosphorylation is the prerequisite of the further differentiation of MDSCs. IRF8 and PU.1 are important transcription factors in macrophage differentiation. Retinoic-acid-related orphan receptor (RORC1), a critical driver of tumor-induced emergency hematopoiesis, regulates MDSCs differentiation by inhibiting negative regulators and promoting positive regulators such as C/EBPβ. Through IRF8 and PU.1, RORC1 promotes MDSCs differentiate into macrophages [34]. Furthermore, GM-CSF and M-CSF drive the differentiation of MDSCs into M2-TAMs [35]. Extracellular ROS promote monocyte differentiation into macrophages and M2 polarization, while high ROS levels induced by NADPH oxidase 2 inhibit MDSCs differentiation [19]. Earlier studies have demonstrated that the differentiation of immature myeloid cells (IMCs) into TAMs and DCs is enhanced in mice cultured after removing ROS [36] (Fig. 2).

Lactate notably impacts the differentiation and development of MDSCs and TAMs. Lactate promotes hypoxia-inducible factor 1 subunit alpha (HIF-1α)-mediated polarization of M2-TAMs and decreases the presence of M1 macrophage markers [37,20]. Zhao et al. identify the Notch/RBP-J pathway, which regulates lactic acid metabolism in myeloid cells to promote M1 macrophage polarization. However, subsequent research indicates that lactate promotes the differentiation of M-MDSCs into PMN-MDSCs, rather than M1 macrophages [38]. In prostate cancer model, the acidic environment induced by lactate favors M2 polarization [39]. In general, the metabolic activities of MDSCs, especially lactic acid metabolism, significantly influence macrophage polarization, driving their shift to TAMs. However, specific mechanisms may differ due to aspects like cell type, tissue environment, and disease state.

### Differentiation of MDSCs into DCs

Dendritic cells are the most functional antigen-presenting cells (APCs) affecting intrinsic and adaptive immune systems. DCs present protein fragments from bacteria, viruses, and tumors on the cell surface major histocompatibility complex I (MHCI) molecules, which are recognized by cytotoxic T lymphocytes and NK cells to initiate adaptive neo-immune responses. DCs are broadly divided into two primary types according to their origin: plasmacytoid DCs (pDCs) and conventional DCs (cDCs). cDCs have unique differentiation and APC properties. The differentiation of cDC1 is dependent on IRF8, which primarily regulates their cell-killing effects on CTLs [40]. Conversely, cDC2 differentiation relies on IRF4, which preferentially activates CD4^+^ T cells [41].

Transcription factors such as STAT3, PU.1, and the IRF family regulate DC differentiation. In the presence of M-CSF, immature monocytes naturally differentiate into mo-Macs. However, when IRF4 is induced by IL-4 and TNF-α, and aryl hydrocarbon receptor (AHR) ligands are bound, these monocytes instead differentiate into monocyte-derived DCs (mo-DCs). The AHR is necessary but not sufficient for DCs differentiation [35,42]. In murine sarcoma models, tumor cells produce retinoic acid, which promotes monocyte differentiation into TAMs not DCs by inhibiting DCs-promoting transcription factor IRF4 [41]. STAT3 activation inhibits myeloid cells from developing into mature forms, which is evident in the expansion of MDSCs and reduced differentiation into DCs in the TME [43]. *In vitro* in bone marrow cultures and tumor-bearing mice, STAT3 inhibition due to VISTA deletion results in decreased differentiation of monocytes into MDSCs and expansion of DCs [14]. The differentiation of DCs is dependent on FMS-like tyrosine kinase 3 (Flt3) and FMS-like tyrosine kinase 3 ligand (Flt3L) [44]. PU.1 promotes Flt3 expression and synergistically regulates the development of DCs. In immune checkpoint blockade (ICB)-resistant non-small cell lung cancer (NSCLC), PU.1 and Flt3 expression are reduced, leading to inhibited differentiation of classical Ly6C^+^ monocytes into DCs/TAMs [45].

**Figure 3:**
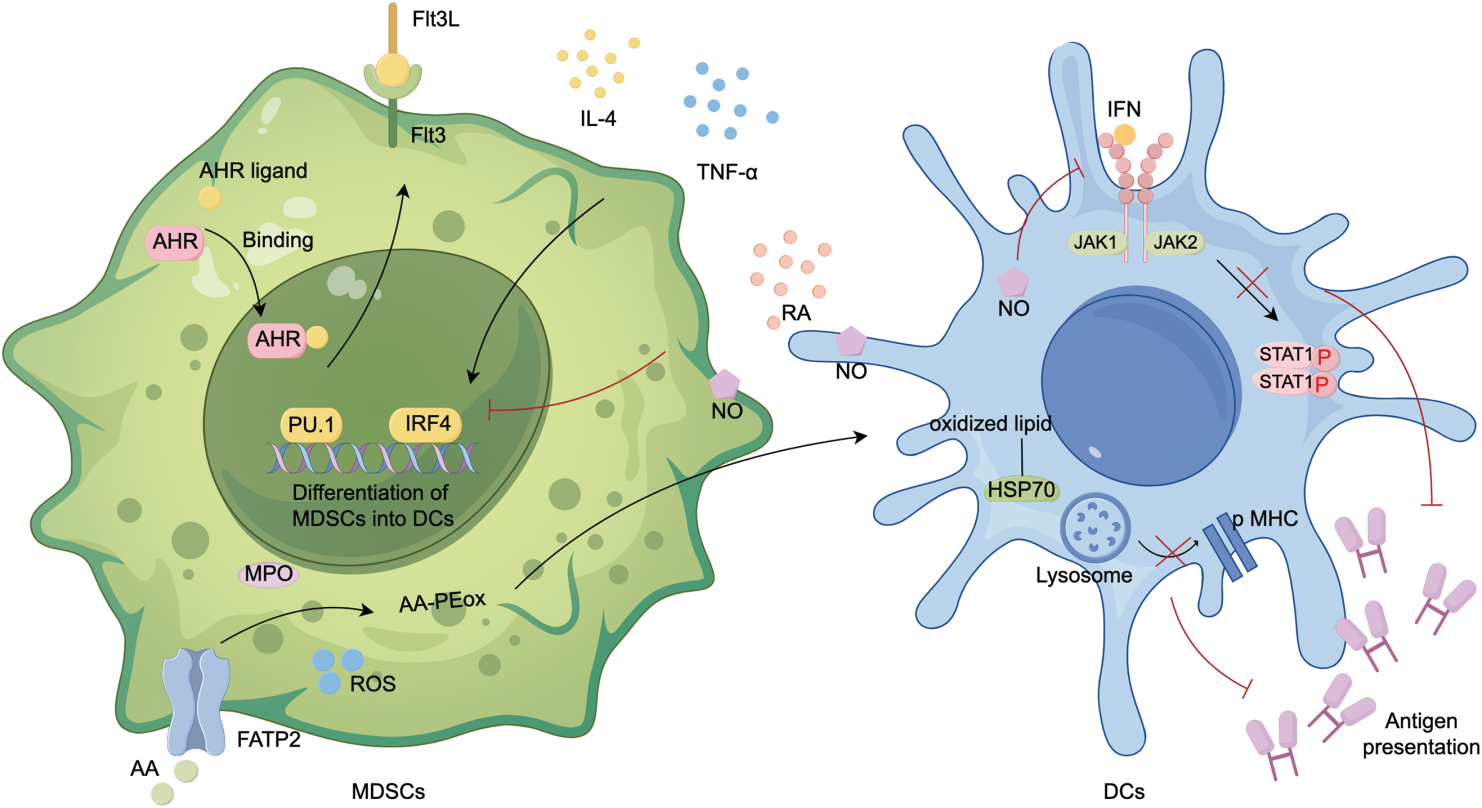
Differentiation of MDSCs into DCs. MDSCs differentiate into DCs under two key conditions: IL-4 or TNF-α activation of IRF4, and AHR binding to ligands. In addition, PU.1 and IRF4 are critical transcription factors that regulate this process. PU.1 promotes Flt3 expression and synergistically regulates the development of DCs. MDSCs are more likely to differentiate into TAMs than DCs, and their primary role is to inhibit DCs antigen presentation: (1) PMN-MDSCs upregulate fatty acid transport protein 2 (FATP2), leading to the uptake of arachidonic acid (AA). In the presence of ROS and myeloperoxidase (MPO), AA undergoes lipid peroxidation to form AA-PEox. These oxidized lipids are then transferred to DCs, where they bind to heat shock proteins (HSPs), preventing the release of peptide-major histocompatibility complex (pMHC) on the cell surface. (2) NO from MDSCs reduces DCs sensitivity to IFN, inhibiting the JAK/STAT1 pathway and preventing DCs from presenting antigens to CD4^+^ T cells. PE, phosphatidylethanolamine; ox, oxidized; created with FigDraw.

DC metabolism in TME is distinguished by a substantial accumulation of neutral lipids linked to the reduced ability to cross-present exogenous antigens, including tumor-specific ones. MDSCs produce abundant lipid peroxidation in the TME. PMN-MDSCs upregulate fatty acid transporter protein 2 (FATP2), and produce oxidized lipids via myeloperoxidase (MPO) and ROS, leading to the cross-presentation function of DCs inhibited [46]. DCs present tumor cell antigens to CD4^+^ T cells through the Jak/STAT1 signaling pathway. NO produced by MDSCs induces nitration of STAT1 at Tyrosine701 and inhibits STAT1 phosphorylation by reducing immune reactivity to IFN [47] (Fig. 3).

In conclusion, MDSCs employ various mechanisms, including producing oxidized lipids or increasing NO, to inhibit DCs from recognizing and presenting antigens. Even when MDSCs differentiate into DCs under the impact of specific cytokines, the antigen presentation capacity of MDSC-DCs will be reduced, which is an adaptation to the immunosuppressive settings [48].

## Plasticity of MDSCs in Cancer Therapy

### Immunotherapy

The advent of immunotherapies like immune checkpoint inhibitors (ICIs), CAR-T cell therapies, and tumor vaccines has markedly improved outcomes for patients with malignant neoplasms. However, drug resistance and suboptimal responses persist. MDSCs are crucial in tumor immunosuppression, though their specific markers remain unclear. The ability to multidirectional differentiation, metabolic reprogramming, and crosstalk with other cells in the TME has spurred therapeutic strategies for direct targeting and combination therapies.

All-trans retinoic acid (ARTA), the major bioactive isoform of retinoic acid [41], decreases immunosuppression and improves antitumor response by reducing the level of ARG1, iNOS, IDO, ROS, and S100A8/A9 to inhibit the function of MDSCs [49]. In addition, ARTA indirectly promotes the accumulation of glutathione by activating the extracellular signal-regulated kinase 1/2 (ERK1/2) pathway, thereby neutralizing the high levels of ROS in MDSCs and facilitating their differentiation [50]. In metastatic melanoma, ATRA significantly reduces PMN-MDSCs and increases HLA^−^DR^+^ myeloid cells, enhancing the sensitivity and efficiency of pembrolizumab [51]. In mouse cervical cancer, ATRA inhibits the accumulation of MDSCs and increases the infiltration of cytotoxic CD8^+^ T cells [52]. In LKB1-deficent murine tumors, ATRA inhibits immunosuppressive MDSCs, enhances T cell cytotoxicity, and increases sensitivity to PD-1 inhibition, thereby increasing tumor sensitivity to ICBs [53]. These findings collectively indicate that ATRA has considerable potential as an immunomodulator and may serve as an adjunctive therapy for immunotherapy.

In metastatic renal cell carcinoma, very small size particle (VSSP), a new immune modulator, lowers the occurrence of PMN-MDSCs and elevates the levels of monocytes and DCs, with high expression of IRF8 and PU.1 [54]. Glutamine metabolism is essential for nucleotide synthesis, amino acid production, and redox balance. Besides IDO inhibition, targeting glutamine metabolism promotes the differentiation of MDSCs into pro-inflammatory TAMs [55]. These suggest that targeting specific molecules or remodeling TME metabolism can promote the differentiation of MDSCs, which is beneficial to enhance theoretical effectiveness. Multiple proceeding or completed clinical trials explore the effects of promoting MDSCs differentiation into TAMs or DCs (Table 1).

**Table 1:** Clinical trials that target MDSCs

Cancer type	Target	Drug	Combination therapy	Phase	Key finding	ClinicalTrials.gov identifier
Melanoma	NA	ARTA	Ipilimumab	II	↓circulating MDSCs, ↑HLA-DR^+^ myeloid cells	NCT02403778
Melanoma	NA	ARTA	Pembrolizumab	I/II	↓total MDSCs and PMN-MDSCs, ↑HLA-DR^+^ myeloid cells	NCT03200847
					ORR 71%; CR 50%	
Oral cavity or oropharynx	PDE5	Tadalafil	NA	NA	↓MDSCs and Tregs in peripheral blood and tumors	NCT00843635
Upper aerodigestive	PDE5	Tadalafil	NA	II	↓circulating MDSCs and Tregs	NCT01697800
Colon	COX2	Celecoxib	FOLFOX	III	↑OS and DFS in patients with PIK3CA gain-of-function mutations (adjusted HR 0.44 and 0.56)	NCT01150045
Urothelial	IDO	Epacadostat	Pembrolizumab	III	ORR 26.2% vs. 11.9% with placebo plus pembrolizumab	NCT03374488
Urothelial	IDO	Epacadostat	Pembrolizumab	III	ORR 31.8% vs. 24.5% with placebo plus pembrolizumab	NCT03361865
Metastatic solid tumors	IDO	Epacadostat	Durvalumab	I/II	ORR 12.0% in the overall phase 2 population	NCT02318277
					ORR 16.1% vs. 4.1% in ICB-naïve patients and patients who have received ICB	
Lung	IDO	Epacadostat	Pembrolizumab	II	No clinical benefit	NCT03322540
					(ORR 32.5% vs. 39.0% with placebo plus pembrolizumab)	
Prostate	CD73	Oleclumab	AZD4635	II	Median rPFS 1.5 months	NCT04089553
					No CRs or PRs	
Colorectal	CD73	Oleclumab	Chemotherapy+bevacizumab	Ib	ORR 61.5%	NCT04068610
					Adverse events 65.4%	
Metastatic solid tumors	ARG1	ARG1 targeting peptide vaccine	NA	I	2 patients (20%) had SD for 4 and 7 months	NCT03689192
Myeloproliferative neoplasm	ARG1	ARG1- and PD-L1-derived vaccines	NA	I–II	↓PD-L1 mRNA expression in peripheral CD14^+^ myeloid cells	NCT04051307
Lung	STAT3	Danvatirsen	Durvalumab	II	MPR 31.3%; pCR 12.5%	NCT03794544
					Failed to significantly enhance the function of CD8^+^ T cells or NK cells	
Lung	XPO1	Selinexor	Docetaxel	I/II	7 patients (22%) had PR, and 18 patients (56%) had SD	NCT03095612
Endometrial	XPO1	Selinexor	NA	III	Median PFS 5.7 months vs. 3.8 months with placebo	NCT03555422

Note: For studies with no reference provided, data are from ClinicalTrials.gov. MDSCs, myeloid-derived suppressor cells; HLA-DR^+^, human leukocyte antigen-DR positive; PMN-MDSCs, polymorphonuclear myeloid-derived suppressor cells; ORR, objective response rate; CR, complete response; Tregs, Regulatory T cells; PDE5, phosphodiesterase type 5; COX2, cyclooxygenase-2; FOLFOX, FOLinic acid, Fluorouracil, and OXaliplatin; OS, overall survival; DFS, disease-free survival; HR, hazard ratio; PIK3CA, phosphoinositide 3-kinase catalytic subunit lpha; IDO, indoleamine 2,3-dioxygenase; ICB, immune checkpoint blockade; rPFS, radiographic progression-free survival; PR, partial response; PFS, progression-free survival; ARG1, Arginase 1; SD, stable disease; STAT3, signal transducer and activator of transcription 3; MPR, major pathologic response; pCR, pathologic complete response; NK cells, Natural Killer cells; XPO1, exportin 1.

Although the particular markers for MDSCs are still unidentified, targeting certain signaling pathways or cytokines has demonstrated distinct effects on MDSCs. IDO1, CD39/CD73, Phosphodiesterase-5 (PDE5), ARG1, COX2 and STAT3 are important therapeutic targets for MDSCs. IDO is a key factor influencing MDSCs function. The IDO/PD-L1 vaccine combined with nivolumab shows sustained and promising efficacy in long-term follow-up, with an overall response rate (ORR) of 80%, and 50% of patients achieving a complete response (CR) [56]. Epacadostat (IDO inhibitor) has shown promise when combined with PD-1 inhibitors in cancers such as melanoma, NSCLC, and urogenital cancers [57–59]. However, in some trials, the results have fallen short. For example, a phase II NSCLC study (NCT03322540) finds no improvement in progression-free survival (PFS) or ORR compared to placebo. These could be due to a small sample size, short follow-up time, and epacadostat doses.

Targeting CD73 or adenosine receptors can block adenosine activity but may also enhance the chemotaxis of MDSCs. For example, in murine pancreatic ductal adenocarcinoma models, AB680 (CD73 inhibitor) increases the chemotaxis of MDSCs through CXCL5 in an AMP-dependent manner [60]. This suggests that chemokine inhibitors are essential to inhibit the aggregation of MDSCs and improve drug response. In a mouse triple-negative breast cancer model, dual blockade of CD73 and TGFβ reprograms the tumor microenvironment by reducing MDSCs and M2 macrophages, while increasing activated Tregs, CD8^+^ T cells, and B cells [61]. Currently, targeting CD73 or adenosine could be a supplement to immunotherapy, but targeting it alone may increase the chemotaxis of MDSCs, leading to limited significant efficacy.

PDE5 inhibitors, such as tadalafil, inhibit MDSCs by lowering levels of iNOS and ARG1. In addition, tadalafil prevents M2 polarization and inhibits polyamine metabolism in TAMs and MDSCs, effectively reversing the immunosuppressive TME and improving the efficacy of ICB in hepatocellular carcinoma [62]. Tadalafil has shown promising clinical results in trials for head and neck squamous cell carcinoma (HNSCC), glioblastoma, and melanoma [63–65]. STAT3 is crucial in MDSCs differentiation, expansion, and activation. Therefore, targeting STAT3 offers a promising avenue for targeting MDSCs. In melanoma model, napabucasin (STAT3 inhibitor) promotes apoptosis of MDSCs in human peripheral blood and mouse bone marrow, thereby enhancing antitumor immunity and prognosis [43]. Furthermore, exportin-1 (XPO1), a key nuclear transport protein, mediates the export of various cargo proteins from the nucleus. Due to its central role in cellular regulation, blocking XPO1-mediated nuclear export has emerged as a promising therapeutic strategy in cancer. IL-6 activates both the JAK/STAT3 and JAK/MAPK signaling pathways in MDSCs. Selinexor (XPO1 inhibitor) exerts a dual effect on MDSCs differentiation. On one hand, it promotes the conversion of MDSCs into neutrophil-like cells with immune-activating properties by intervening in the ERK1/2 signaling within the MAPK pathway. On the other hand, it decreases the expression of immunosuppressive markers and reduces the immunosuppressive function of MDSCs [66]. Selinexor has demonstrated efficacy in clinical trials (NCT03147885, NCT03555422, NCT02269293), particularly in hematologic malignancies and advanced solid tumors (e.g., endometrial and ovarian cancers), especially when used in combination therapies.

In conclusion, modulating signaling pathways to remove MDSCs is increasingly central to cancer immunotherapy. The new target EP2/4 is currently being evaluated in several clinical trials (NCT06129604, NCT05940571). There are active clinical trials targeting MDSCs, and a deeper understanding of their role in combination therapies is needed (Table 1).

### Chemotherapy

Chemotherapy kills tumor cells and surrounding cells, including MDSCs. Moreover, some chemotherapeutic drugs trigger cancer cells to release signaling molecules that activate APCs and induce immunogenic cell death (ICD) [67]. Chemotherapeutic drugs suppress MDSCs, enhance T-cell responses, and improve the immunosuppression of the TME. Cisplatin represses MDSCs expansion by suppressing STAT3/COX-2 signaling and enhances T-cell responses in melanoma and HNSCC patients [68]. Gemcitabine and celecoxib (COX2 inhibitors) induce apoptosis in MDSCs (9.9% and 6.9%), with apoptosis rates increasing to 21.0% after combined application [67]. This shows that chemotherapy and immunotherapy have synergistic effects in depleting MDSCs.

However, the immunosuppressive nature of the TME decreases such immune response. Chemotherapeutic agents can also generate tumor resistance by upregulating MDSCs. Cisplatin, oxaliplatin, and adriamycin can increase oxidized 1-palmitoyl-2-arachidonoyl-sn-glycero-3-phosphocholine (oxPAPC) release in tumors. In WT LL2-bearing mice, a significant up-regulation of MDSCs in the TME is observed after oxPAPC injection [69]. In a ovarian cancer mouse model, carboplatin increases M-MDSCs and M2 TAMs, while carboplatin-paclitaxel do not reveal any immunological alterations [70]. Nevertheless, Paclitaxel resistance hinders its effectiveness in breast cancer. Combing with Rg3-based liposomes not only targets tumor cells but also repolarizes pro-tumor M2 macrophages to the antitumor M1 phenotype and suppress MDSCs, thereby remodeling the TME [71]. Different impacts of MDSCs from chemotherapeutic drugs may result from tumor-specific immunobiology and population immune heterogeneity.

### Radiotherapy

Radiotherapy and ICD in various cancers are aimed directly at inducing DNA damage and tumor cell apoptosis in the TME [72]. The YTHN6-methyladenosine RNA binding protein 2 (YTHDF2), a major m6A RNA-binding protein, is deleted after ionizing radiation (IR) in mouse M-MDSCs. This results in M-MDSCs differentiating more into M1 macrophages and enhanced antitumor immunity [73]. Radiotherapy has contradictory effects on the suppression and promotion of tumor progression. Local irradiation increases systemic MDSCs, enhances immune suppression, and promotes tumor metastasis [74]. STING activation by radiation promotes the recruitment of MDSCs through the CCR2 pathway and enhanced antitumor immunity [75]. Together, extrinsic radio resistance is associated with MDSCs. MDSCs are a critical factor in the generation of immune tolerance. Targeting MDSCs is beneficial to improving therapeutic efficacy. However, the mechanism by which radiotherapy affects MDSCs, and the clinical benefit of combination therapy still need further experimental studies to validate.

### Targeted therapy

Targeted therapy aims to inhibit tumor progression by targeting specific genetic mutations or molecules while minimizing side effects. Targeted therapies can modify the function and phenotype of MDSCs to improve antitumor immunity, especially in combination with ICIs. For example, demethylation inhibitor decitabine promotes MDSCs differentiation into M1 macrophages by upregulating IRF7. Decitabine also activates innate immune pathways, including NOD-like signaling, thereby enhancing antitumor immune responses [76]. Guadecitabine is a dinucleotide prodrug of decitabine that inhibits DNA Methyltransferase 1 (DNMT1) and induces hypomethylation. In a breast cancer mouse model, guadecitabine eliminates most MDSCs and induces a subset to express APC markers and co-stimulatory molecules like major histocompatibility complex II (MHCII) and CD80/86 [77]. Furthermore, histone deacetylase inhibitors CN133 inhibits the recruitment of PMN-MDSCs by downregulating genes associated with their migration, which restores a positive immune environment and significantly enhances the efficacy of PD-1 therapy in prostate cancer [78]. In a mouse lung metastasis model, low doses DNA methyltransferase, histone deacetylase inhibitors, 5-azacytidine and entinostat promote the differentiation of MDSCs into macrophage, disrupting the pre-metastatic immunosuppressive environment [79]. These findings highlight that epigenetic mechanisms can reshape MDSCs function and improve immune responses in the TME.

In addition, Bruton’ s tyrosine kinase (BTK) inhibitor ibrutinib, increases the frequency of M-MDSCs and reduces chemokines such as CCL2, CCL3 involved in MDSCs recruitment [80]. However, acalabrutinib (another BTK inhibitor) has shown limited efficacy in pancreatic cancer patients. While it reduces PMN-MDSCs and induces T cell activation, ORR and disease control rate (DCR) for monotherapy were only 0% and 14.3%. BTK inhibitors may require combination with other immune therapies to achieve optimal therapeutic outcomes [81]. Sunitinib (multi-target tyrosine kinase inhibitor) has been associated with MDSCs expansion and immune resistance. In mouse models, the combination of sorafenib and tazemetostat (enhancer of zeste homolog 2 inhibitor) significantly reduces MDSCs and Treg populations, promotes T cell infiltration, and reverses immune resistance in HNSCC [82].

In summary, the plasticity of MDSCs in targeted therapy represents a promising avenue for improving antitumor immunity. By remodulating MDSCs function and phenotype, these therapies offer potential to enhance the efficacy of immune therapies and reverse immune resistance, providing new insights for future cancer treatment strategies.

## Discussion and Conclusion

The plasticity of MDSCs plays a crucial role in tumor immune evasion. In the TME, MDSCs respond to a complex network of cytokines and signaling pathways that regulate their differentiation, phenotype, and function. Especially, this plasticity enables MDSCs to differentiate into various immune cell types, such as TAMs and DCs, depending on the specific conditions of the tissue or microenvironment. Hypoxia and acidic environment, along with the downregulation of STAT3 activity, promote MDSCs differentiation into TAMs, while MDSCs differentiation into DCs is less frequent and associated with a diminished antigen-presenting capacity. Additionally, metabolic factors, including glutamine metabolism and ROS levels, along with epigenetic modifications such as m6A methylation, significantly influence MDSCs plasticity and their immunosuppressive function. Despite the therapeutic potential of targeting MDSCs to overcome tumor immune evasion, the clinical translation of such therapies faces several challenges. These include the lack of specific targeting markers, the complexity of the TME, and immune resistance. Therefore, future research should focus on further elucidating the molecular mechanisms driving MDSCs plasticity, particularly the signaling and metabolic pathways involved, as well as developing more precise targeting strategies. The rise of single-cell analysis and high-throughput sequencing technologies offers promising avenues for a deeper understanding of MDSCs heterogeneity and the identification of novel therapeutic targets. In conclusion, MDSCs plasticity is a critical driver of tumor immune escape and a promising target for therapeutic intervention. By precisely modulating MDSCs differentiation and function, it may be possible to overcome current treatment limitations and enhance the efficacy of immune therapies. Future research should aim to translate these findings into clinical strategies to improve cancer treatment outcomes.

## Data Availability

Not applicable.
